# The mediating role of early maladaptive schemas in the relationship between attachment styles and loneliness

**DOI:** 10.1186/s40359-023-01172-9

**Published:** 2023-04-27

**Authors:** Kosar Jalilian, Khodamorad Momeni, Hashem Jebraeili

**Affiliations:** Department of Psychology, Faculty of Social Sciences, Razi University, Kermanshah, Iran

**Keywords:** Loneliness, Object attachment, Early maladaptive schemas, Iranian people, University students

## Abstract

**Background:**

As with the increasing prevalence of loneliness among college students, it seems necessary to investigate the early grounds of its formation. Therefore, the present study was conducted to examine the relationship between attachment styles and loneliness through the mediating role of early maladaptive schemas (EMS).

**Methods:**

This research was correlational, of structural equations modeling (SEM) type. The statistical population included all the college students of the universities of Kermanshah in the academic year 2020–2021, of whom 338 were selected using convenience sampling. The measures used in this study included DiTomasso et al.’s social and emotional loneliness of adults, Hazan and Shaver’s adult attachment, and Young’s schema scales. For data analysis, Pearson’s correlation coefficient and SEM were used in Lisrel 8.8 and SPSS-22 software.

**Results:**

The results illustrated that the hypothesized model of the study has a good fit in the studied sample. It was also found that both the avoidant and ambivalent attachment styles are related to loneliness through two EMS of disconnection-rejection and other-directedness.

**Conclusions:**

Based on the findings, measures are recommended to increase information regarding the basic and underlying factors affecting loneliness for therapists and psychological specialists.

## Background

Adulthood and youth are the period of growth and learning. However, many college students experience the transition to adulthood as a challenge to their emotional health [1]. college students do not talk about their feelings and emotions easily, and therefore feeling lonely is a common problem among them [2]. In recent decades, the prevalence of loneliness in the general population has become more alarming. As such, studies indicate that 10.5% of people experience some degree of loneliness [3]. Besides, in Iran, Alaviani et al.’s [4] study on college students revealed that the prevalence of moderate loneliness in college students is 31.6% and that of severe loneliness is 5.5%.

Loneliness is an unpleasant feeling that occurs when people perceive their network of social relationships broken, because they have fewer relationships than they would like, or because they think the quality of their existing relationships is insufficient [5]. Many studies have investigated this construct and its consequences. For example, loneliness has been found to have a positive relationship with deficits in cognitive functions [6, 7], and psychotic [8], and a negative relationship with life satisfaction [9, 10] and optimism [11]. Loneliness has been highlighted as a growing public health issue in developed countries [12]. Therefore, researchers have investigated the role of many variables in predicting this construct. One of the prominent theories in predicting the loneliness appears to be the basic interpersonal relationships of people. Post-Freuidian psychoanalysts believed that the loneliness originates from narcissism, childhood hostility, failure to satisfy childhood needs [13] and the lack of early attachment Figs. [14]. Therefore, attachment and care styles are considered as the main components of interpersonal relationships, and according to previous studies, they can play a decisive role in predicting loneliness [15–18].

Adult attachment styles refer to a people’s persistent desire to try to be close to and contact one or more special people who provide them with a sense of physical and psychological safety and security [19]. Hazan and Shaver [20] divided adult attachment styles into three categories of secure, avoidant and ambivalent. People with secure attachment style are comfortable in intimate relationships and describe their attachment figures as warm people. Ambivalent people have a strong desire to communicate, but at the same time they worry about rejection and consider acceptance from others as a necessary condition for feeling positive about themselves. Avoidant people seek self-reliance, and when they are likely to be rejected by others, they tend to maintain positive self-images by denying the need for attachment, while they have negative attitudes towards others [20].

Many studies have investigated the relationship between attachment styles and loneliness. However, recently, the existing studies in this field have focused more on the nature of this relationship. In fact, these studies seek to know how pathway of attachment styles are related to loneliness. For example, studies have shown that attachment styles through mediating variables such as social support [21], basic psychological needs [22], general belongingness [23] and the feeling of inferiority [24] are related to the loneliness. One of the important mental frameworks raised in recent studies regarding loneliness and attachment is thought to be early maladaptive schemas (EMS).

EMS, Young [25] believes, arises due to the failure to satisfy a child’s basic emotional needs such as the need for secure attachment. Meanwhile, Bowlby’s [26] attention to internal working models overlaps with the emphasis on EMS. Schemas are dysfunctional internal patterns that direct children’s responses to attachment figures and their coping styles. Using these patterns, children predict the behavior of attachment figures and prepare to respond to them [27]. EMS are deep-seated patterns about self and others that are dysfunctional. These schemas are made up of memories, emotions, cognitions and physical feelings. They are formed during childhood or adolescence and become more complex throughout life and affect future life experiences [25]. Young et al. [25] listed eighteen EMS in five domains. Two widely used domains are those of disconnection-rejection domain (including the schemas of emotional deprivation, abandonment/instability, mistrust/abuse, social alienation/rejection, defectiveness/shame) and the other-directedness domain (including the schemas of subjugation, self-sacrifice, approval-seeking/recognition-seeking). EMS predict interpersonal maladjustment and explain many psychological and personality disorders [28]. Sullivan [29] emphasizes the need to establish a relationship with others because it is rooted in basic needs and puts forward the inadequacy in fulfilling the need for intimacy with others as loneliness. One of the factors in the emergence of psychological distress and loneliness is likely to be EMS, because EMS usually originate from the failure to meet basic needs, especially emotional needs, in childhood, and impose themselves on later life experiences [30]. Previous studies indicate a significant relationship between EMS and loneliness [31–34]. Menz et al.’s [32] evinced that EMS are capable of mediating the relationship between anxious and avoidant attachment dimensions with psychopath symptoms. According to this research, the schemas of disconnection-rejection and other-directedness play a mediating role in the relationship between anxious attachment and psychopathology, and the disconnection-rejection schema play a mediating role in the relationship between avoidant attachment and psychopathology. Moreover, previous studies indicated a significant relationship between attachment styles and EMS [35–37].

**Fig. 1:**
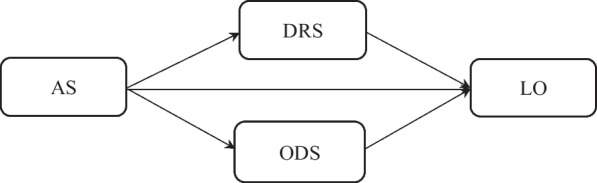
Hypothesized model of the relationship between attachment styles and loneliness through EMS

Although the correlation of EMS with loneliness has been shown and the highlighted role of attachment styles in loneliness and EMS is clear in the research literature, to the best of our knowledge, so far, the relationship between these variables as a structural model has not been investigated in college students and this gap is clearly evident in the research literature. In addition, as loneliness is an ever-increasing issue among college students and can cause them to face some educational problems and daily challenges, it is crucial to conduct studies in this field of inquiry in order to determine the factors, such as attachment styles, involved in creating feelings of loneliness. Because attachment styles are formed in first years of people’s life, they grow with them and play a predominant role in the emergence of deep internal working model and early schemas regarding themselves and their relations with significant others. Therefore, a better understanding of the role played by attachment styles in the formation of early schemas and the interrelation between these two variables in college students’ feelings of loneliness can provide a deep understanding about loneliness for psychotherapists working in this field. Therefore, conducting studies in this field to determine the factors involved in creating loneliness reveals many hidden aspects and creates a better understanding of this issue for psychotherapists working in this field. Therefore, it seems necessary to carry out this study in order to solve the research gaps in this field and also to better understand the factors affecting the formation of loneliness. Hence, the present study aimed to investigate the relationship between attachment styles and loneliness with the mediating role of EMS. The hypotheses of this study were that [1] attachment styles are related to loneliness through the schema of disconnection-rejection, and [2] attachment styles are related to loneliness through the other-directedness schema. The hypothesized model of this study is shown in Fig. 1.

## Methods

### Research design and participants

The method of the current research was correlational of structural equation modeling type. The statistical population of the present study included all the college students of Kermanshah, Iran in the academic year 2020–2021, of whom 338 (256 women and 82 men) were selected through convenience sampling. It is worth explaining that Stevens [38] considered 15 cases for each predictor variable in multiple regression analysis with the standard least squares model as a good rule of thumb. Based on this issue, it can be stated that as SEM is completely related to multivariate regression in some aspects, the number of 15 items for each measured variable in SEM is not unreasonable. Loehlin [39] stated that for models with two or four factors, the researcher should plan on collecting at least 100 cases or more, say 200 cases. Therefore, in order to determine the sample size, the calculation based on the number of components of the research variables was used. Taking into account the number of samples of 15 for each component and considering that the current research included 13 components, the minimum number of samples needed to conduct the study was 195. However, since there is always the possibility of participants dropping out and having questionnaires distorted, and considering Loehlin’s recommendation to have a sample of more than 200, we tried to consider more than 300 participants as the study sample, and finally 338 participants completed our questionnaires. The inclusion criteria were [1] studying in one of the universities of Kermanshah at any stage and [2] the desire to participate in the study, while the exclusion criterion was the experience of bereavement of a loved one in the previous three months.

### Measures

All the questionnaires applied in the current study were built and standardized in the countries other than Iran. However, all of them had been already standardized in Iran by other researchers using confirmatory factor analysis, the results of which are presented below for each questionnaire.

### Social and emotional loneliness scale for adults (SELSA-S)

This scale was designed and prepared by DiTomasso et al. [40]. This scale includes 14 items and its three subscales include romantic loneliness, family loneliness and social loneliness. For each item, there is a 5-point Likert scale (1 = completely disagree to 5 = completely agree). The minimum possible score is 14 and the maximum is 70, with the average of 42. A lower score means less loneliness in adults. Ditommaso et al. [40] reported its Cronbach’s alpha coefficient between 0.87 and 0.90, which indicates the appropriate internal consistency of the scale. They also reported that there is a significant correlation between SELSA-S with the total score of Russell’s [41] revised loneliness scale. In Iran, confirmatory factor analysis of the questionnaire, done by Jowkar and Salimi [42], showed that the three-factor structure has a good fit with the data. Cronbach’s alpha coefficients for the subscales of romantic, social, and family loneliness were equal to 0.92, 0.84, and 0.78, respectively [42]. In the present study, the reliability of this questionnaire based on Cronbach’s alpha for subscales of romantic, family and social loneliness, was 0.91, 0.89 and 0.81, respectively.

### Adult attachment questionnaire (AAQ)

This questionnaire, created by Hazan and Shaver [20], distinguishes three attachment styles of secure, avoidant and ambivalent. This questionnaire has 21 items scored on a 5-point Likert scale (1 = none to 5 = very high). The minimum possible score is 15 and the maximum is 35, with the average of 75. A higher score in each of the subscales means that the characteristics of that type of attachment are high in the person and vice versa. Hazen and Shaver found the reliability of this questionnaire to be 0.81 using retest method with a time interval of two weeks, and 0.78 with Cronbach’s alpha method. In Iran, Cronbach’s alpha coefficient of each of the security, avoidant and ambivalent subscales for a student sample was 0.74, 0.72, 0.72 respectively [43]. In the study of Parvizi and Sadeghi [44] this questionnaire was subjected to confirmatory factor analysis, and the results revealed the good fit of the model with the data. In the present study, the reliability of this questionnaire based on Cronbach’s alpha for secure, ambivalent and avoidant subscales was obtained to be 0.81, 0.86, and 0.89, respectively.

### Young schema questionnaire (YSQ-SF)

This questionnaire, created by Young [45], has 75 items to identify 15 EMS in the form of five domains including emotional deprivation, abandonment/instability, mistrust/abuse, social isolation/alienation, defectiveness/shame, failure, dependence/incompetence, vulnerability to harm and illness, enmeshment/undeveloped self, subjugation, self-sacrifice, emotional inhibition, unrelenting standards/hypercriticalness, entitlement/grandiosity and insufficient self-control/self-discipline. Questions are scored on a 6-point Likert scale. A higher average in each schema means that schema is active in the person. Smith et al. [46] indicated that the Cronbach’s alpha coefficient in the non-clinical population for the subscales of this questionnaire was between 0.5 and 0.82. Using confirmatory factor analysis, Ghiasi et al. [47] showed that the factor structure of the questionnaire has a good fit with the data. They also found that Cronbach’s alpha coefficients for the disconnection-rejection and other-directedness domains were 0.90 and 0.76, respectively. In this research, only two domains of disconnection-rejection (including schemas of emotional deprivation, abandonment/instability, mistrust/abuse, social isolation/alienation and defectiveness/shame) and other-directedness (including schemas of subjugation and self-sacrifice) were used. In the present study, the reliability of this questionnaire was obtained based on Cronbach’s alpha for the subscales of disconnection-rejection and other-directedness to be 0.86 and 0.81 respectively.

### Procedure

Having obtained the necessary permits to conduct this research from the competent authorities of Razi University of Kermanshah, we carried out the steps of data collection. In these stages, the necessary questionnaires were designed in the form of online questionnaires. The manner of preparing online questionnaires is that the questions of a questionnaire along with the rating scales of any question are entered in a website which provide data collection services in Iran (the website utilized in the current study was digit.kums.ac.ir). Then, the website creates a link through which people can achieve the questionnaire. Therefore, when people click on the mentioned link, they can enter the questionnaire webpage and answer the questions. Due to the simultaneous implementation of this study with the severe peak of the Covid-19 virus in Iran, the link of the questionnaire was provided to the college students studying in the universities of Kermanshah province, Iran through e-mail or social networks. In this sense, they could click on the link which sent them to the webpage of the questionnaires for participants to answer the questions. Before the participants complete the questionnaires, the objectives of the research were clarified for them. In this regard, explanations were provided regarding the disclosure of identity information, confidentiality and privacy of the participants, and their informed consent to participate in the research was obtained. First, the participants answered preliminary questions that included the inclusion criteria, and those who met the inclusion criteria were allowed to access the main questionnaires. In addition, in the online implementation of this research, the questionnaires were designed in such a way that the participants could send the questionnaire if they answered all the questions. Therefore, there were no missing data in the study. Finally, by examining 338 questionnaires completed by the statistical sample, after discarding 19 participants (due to the presence of outliers), the information obtained from 319 people was analyzed as the final sample of the research. The data obtained from the questionnaires were analyzed using the Pearson’s correlation coefficient method and structural equation modeling (SEM) in Lisrel 8.8 and SPSS-22 software.

## Results

The average age [18–53 years] of the participants was generally 25.09 (SE = 5.02). Among participants, 282 were single (83.4%) and 56 married (16.6%). Out of all participants, 10 (3%) reported their education as associate of art, 215 (63.6%) as bachelor, 104 (30.8%) as master and 9 (2.7%) as PhD student.

Before performing the analysis, the assumptions of SEM including normality of distribution, independence of errors and multiple collinearity were checked. To assume the normality of the research variables, the skewness and kurtosis of the distribution of scores were used, and the results showed that the distribution of the scores of all variables is normal (the range of distribution was between + 1 and −1). Durbin-Watson test was used to check the independence of errors, the results of which showed no correlation between errors (DW = 1.98, the range between 1.5 and 2.5 is acceptable). Variance Inflation Factor (VIF) and Tolerance were used to check the multiple collinearity between the predictor variables, and the results showed that there is non-collinearity between the variables (the range of VIF was less than 5 and the tolerance was higher than 0.1). The results related to these assumptions are presented in Table 1 along with the average and standard deviation of the variables.

**Table 1:** The mean, standard deviation, and information regarding assumptions of SEM

Variable	Minimum	Maximum	M	SD	Skewness	Kurtosis
AS						
SE	8	25	15.95	3.09	0.075	0.052
AM	5	23	12.10	3.74	0.383	0.058
AV	6	28	15.35	3.87	0.328	0.254
DRS	20	108	49.5	16.13	0.839	0.613
ED	4	24	10.97	5.17	0.557	0.564
AB	4	24	11.7	4.81	0.649	0.240
MA	4	24	10.6	4.48	0.657	0.036
SI	4	24	9.53	4.31	0.808	0.025
DS	4	20	6.95	3.47	1.306	0.980
ODS	6	33	14.62	5.25	0.683	0.195
SU	3	17	5.31	2.92	1.518	1.758
SS	3	18	9.31	3.52	0.301	-0.554
LO	11	54	26.06	8.74	0.406	-0.073
RO	3	15	8.89	1.4	0.103	-1.202
FA	4	20	8.06	3.7	0.944	0.38
SO	4	20	9.14	3.70	0.716	0.032

AB = Abandonment/Instability, AM = Ambivalent, AS = Attachment Styles, AV = Avoidant, DRS = Disconnection-rejection Schema, DS = Defectiveness/Shame, ED = Emotional Deprivation, FA = Family, LO = Loneliness, MA = Mistrust/Abuse, ODS = Other- Directedness Schema, RO = Romantic, SI = Social Isolation/Alienation, SO = Social, SS = Self-Sacrifice, SU = Subjugation

Another assumption is the presence of a linear relationship between independent and dependent variables, which was investigated with Pearson’s correlation coefficient, the results of which are reported in Table 2.

**Table 2:** The correlation coefficient between the variables

Variable	LO	RO	FA	SO
SE	−0.196**	−0.082	0.031	0.268**
AM	0.118*	0.069	0.172**	0.03
AV	0.375**	0.287**	0.283**	0.383**
DRS	0.505**	0.346**	0.413**	0.394**
ED	0.571**	0.436**	0.447**	0.416**
AB	0.086**	0.027	0.091	0.082
MA	0.268**	0.203**	0.213**	0.194**
SI	0.456**	0.288**	0.375**	0.381**
DS	0.466**	0.303**	0.388**	0.374**
ODS	0.148**	0.071	0.177**	0.094**
SU	0.226**	0.061	0.282**	0.183**
SS	0.033	0.055	0.029	0.012**

*P < 0.05, **P < 0.01

AB = Abandonment/Instability, AM = Ambivalent, AS = Attachment Styles, AV = Avoidant, DRS = Disconnection-rejection Schema, DS = Defectiveness/Shame, ED = Emotional Deprivation, FA = Family, LO = Loneliness, MA = Mistrust/Abuse, ODS = Other-Directedness Schema, RO = Romantic, SI = Social Isolation/Alienation, SO = Social, SS = Self- Sacrifice, SU = Subjugation

**Fig. 2:**
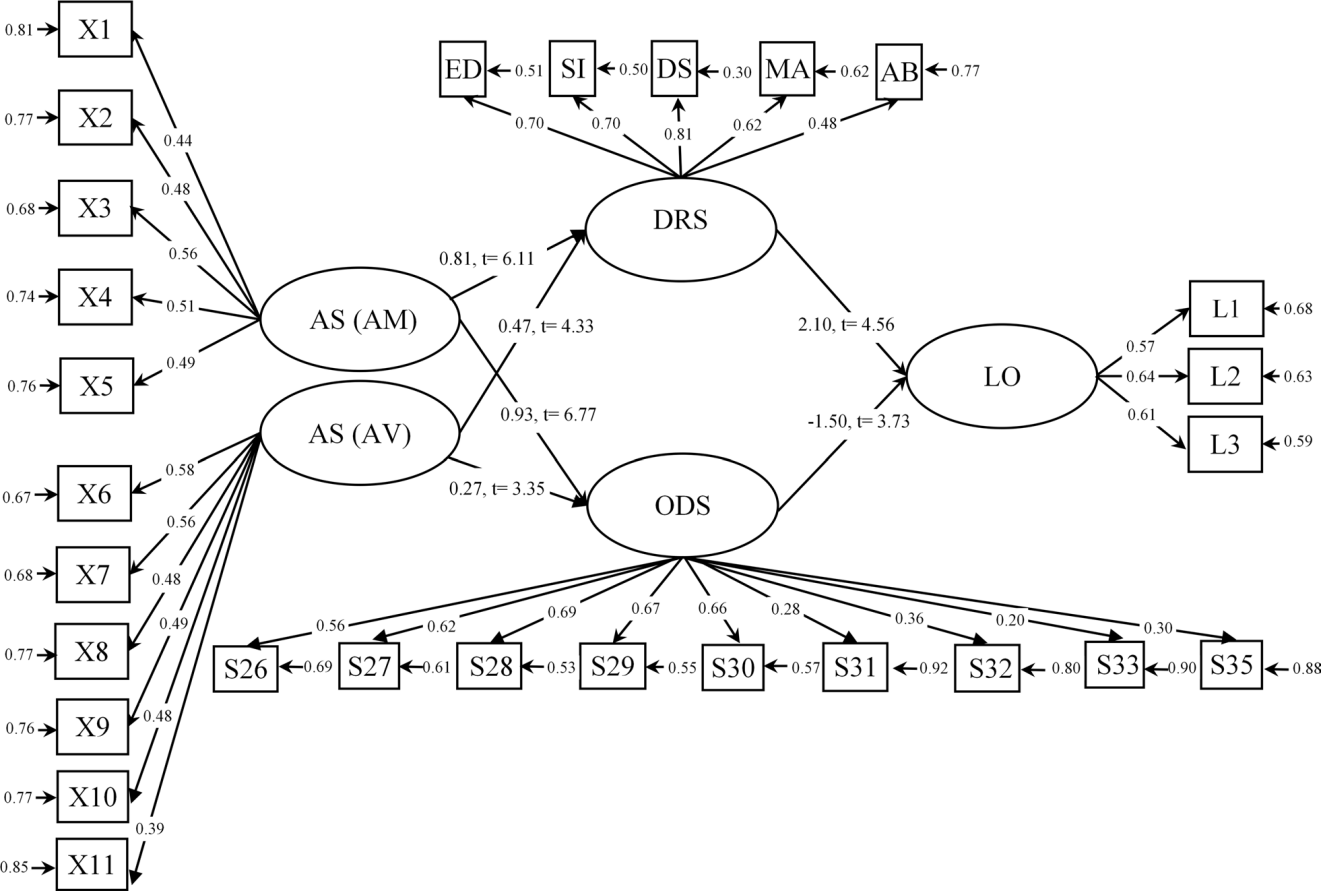
the model of the relationship between attachment styles and loneliness through EMS

Table 3 shows the fit indices of the proposed and modified research model. According to the contents of Table 3, to determine the adequacy of the proposed model with the data, a combination of goodness-of-fit indices such as chi-square value (*χ*^2^), normalized chi-square measure (the ratio of chi-square to degrees of freedom), goodness-of-fit indices (GFI), normalized fit (NFI), comparative fit (CFI) and root mean square error of approximation (RMSEA) were used. The final model of the study can be seen below in Fig. 2.

**Table 3:** Fit indices for the developed model

Model fit indices	X^2^	df	X^2^/df	GFI	IFI	CFI	RMSEA
Obtained values	917.96	334	2.74	0.93	0.92	0.91	0.075

According to Table 3, the amount of fit indices such as incremental fit index (IFI = 0.92), comparative fit index (CFI = 0.91), normalized fit index (NFI = 0.91) as well as root mean square of the approximate residuals (RMSEA = 0.075) indicates a very good fit of the model with the data. Other fit indices such as goodness of fit index (GFI = 0.93) and adjusted goodness of fit index (AGFI = 0.92) are also acceptable. Table 4 shows the parameters related to the direct effects of variables on each other in the proposed model.

**Table 4:** Coefficients of the model of the relationship between AS (AM and AV) and EMS (DRS and ODS) through EMS and LO

Direct path	Regression coefficient	Statistic- t
AM → DRS	0.81	6.11**
AM → ODS	0.93	6.77**
AV → DRS	0.47	4.33**
AV → ODS	0.27	3.35**
DRS → LO	2.1	4.56**
ODS → LO	−1.5	−3.73**

**P < 0.01

AM = Ambivalent, AS = Attachment Style, AV = Avoidant, DRS = Disconnection-rejection Schema, EMS = Early Maladaptive schema LO = Loneliness, ODS = Other- Directedness Schema

As the results in Table 4 show, the direct paths are significant and the path of secure attachment to the schemas of disconnection-rejection, other-directedness and loneliness is removed. Also, the Sobel test was used to investigate the mediating role of disconnection-rejection and other-directedness schemas in the relationship between attachment styles and loneliness, the results of which are reported in Table 5.

**Table 5:** Results of investigating the mediating role of DRS and ODS in the relationship between AS (AM and AV) and LO

Variables	P	Sobel’s test (z)
AM → DRS → LO	*P* < 0.001	7.44
AM → ODS → LO	0.01	2.6
AV → DRS → LO	*P* < 0.001	5.46
AV → ODS → LO	0.12	1.51

AM = Ambivalent, AS = Attachment Style, AV = Avoidant, DRS = Disconnection-rejection Schema, LO = Loneliness, ODS = Other-Directedness Schema

Referring to Table 3, the proposed model of the research has good fit indicators. The root mean square index of the approximate residuals (RMSEA = 0.075) indicates a good fit of the model with the data. Therefore, the proposed model has a good fit in the target sample. In the meantime, secure attachment style could not play a role in the proposed model and was removed from the equations for better modeling, and two attachment styles, avoidant and ambivalent, remained in the model. According to the information in Table 5, the significance of the Sobel test indicates the significance of the mediation role of the disconnection-rejection schema and the other-directedness in the relationship between ambivalent attachment and loneliness. Avoidant attachment, however, only has a significant relationship with the loneliness through the schema of disconnection-rejection, and this is not significant through the mediating role of other-directedness schema.

## Discussion

The present study was conducted with the aim of investigating the relationship between attachment styles and loneliness with the mediating role of EMS. The results showed that the assumed model of this study has a good fit in the studied sample. This means that, on the one hand, people with ambivalent attachment style, due to more experience of disconnection-rejection and other-directedness schemas, experience more loneliness. On the other hand, people with avoidant attachment, due to more experience of disconnection-rejection schema and other-directedness, experience more loneliness.

The first hypothesis of this research, ambivalent and avoidant attachment styles have a positive relationship with loneliness through the disconnection-rejection schema, was confirmed based on the findings. In other words, people with ambivalent and avoidant attachment style have a more intense disconnection-rejection schema, which in turn is related to feeling more alone. Although no study has investigated this path independently, this study, in terms of the relationships that form the above path, that is, the relationship between ambivalent attachment and disconnection-rejection schema [36, 48, 49], the relationship between avoidant attachment and disconnection-rejection schema [18, 50, 51] and the relationship between disconnection-rejection schema and loneliness [31, 32, 52–54] is in line with previous studies. In explaining this finding, Bowlby’s attachment theory can be considered in which Bowlby [14, 26] emphasized that early childhood communication shapes people’s attachment style and affects their view of themself, others, and the way interpersonal communication is organized. According to this theory, the impact of attachment quality continues throughout life and explains individual differences in the field of methods of dealing with internal disturbance and regulating interpersonal relationships. If taking care of the child is not accompanied by responsiveness and availability, insecure attachment will be formed in the child and his internal working models or mental schemas will be shaped on the fact that others are unattainable and untrustworthy. People with an ambivalent insecure attachment style are clearly characterized with low self-confidence. These people associate their important relationships with low levels of satisfaction, commitment, trust, and dependence, but they are not indifferent to their attachment figure and have a greater fear of separation and rejection from their attachment figure. People with ambivalent attachment are often reluctant to get close to others, and if they do get close, they are constantly worried that the emotional partner or people around them will not reciprocate their feelings, thereby resulting in separation. These people are very disturbed, especially after the end of the relationship. The schema of disconnection-rejection also fuels this confusion. The disconnection-rejection schemas usually arise in families that are emotionless, cold, skimp, recluse, inflammable, unpredictable, or misbehaving. According to this schema, people who have ambivalent attachment do not consider the important people in their lives to be reliable and are worried every moment that they will lose them, be rejected by them, be hurt and neglected, and finally these schemas increase the loneliness in people [25]. If people under the influence of avoidant insecure attachment also experience the schema of disconnection-rejection, they are afraid of intimacy, and are constantly worried about being rejected or neglected, being unloved and being misunderstood and unsupported by others due to their lack of trust in others and finding the important people of their life as unstable and unpredictable ones. This path actually increases the loneliness in these people. Avoidant attachment is a dangerous and damaging factor for relationships, and it leads to the possibility of breaking up and separating relationships, and then these people feel [55].

The second hypothesis of the research that ambivalent and avoidant attachment styles are related to loneliness through the other-directedness schema was partially confirmed based on the obtained results. In other words, just people with ambivalent attachment experience an active other-directedness schema, which leads to a decrease in their loneliness. Although so far no study has investigated this path independently, this study, in terms of the relationship of ambivalent attachment with the other-directedness schema [56–58], the relationship of avoidance attachment with other-directedness schema [32, 49, 59, 60] and the relationship of the other-directedness schema with the loneliness [33, 34, 61] is consistent with previous studies. In explaining these results, it can be stated that according to attachment theory, relationship satisfaction during childhood affects later relationships. Having disturbed attachment to parents, teenagers and young people due to mental models or negative self-images towards themselves or others have a cognitive representation that they do not deserve love or that others are not reliable. These assumptions and expectations about oneself and others can affect a person’s ability to establish and develop intimate and close emotional relationships and reduce the quality and intimacy of interpersonal relationships [62]. Insecure ambivalent individuals, despite their mistrust and anxiety about losing significant others, also desire intense intimacy [63]. This finding is consistent with the attachment theory in that ambivalent people need intimacy and feel lonely when they need closeness. Also, these people fear loneliness, which prevents them from leaving the relationship [64]. Ambivalent people do not trust others in their relationships, but they become very dependent in the hope that their needs will be met. Their extreme need is to be loved by the other person and they do their best in the hope of obtaining this wish [65]. Schemas are created and developed on the internal working model of attachment theory. Therefore, attachment figures can be conceptualized as cognitive schemas for relationships that are formed in response to caregiver-child experiences and subsequent interpersonal relationships [66]. The schemas of the other-directedness characterized by the two schemas of subjugation and self-sacrifice, reflect an external source of control in the individual. These two schemas may cause problems by ignoring the emotional needs of the person and focusing on getting the satisfaction of others. Especially, one of the important consequences of the sacrifice schema is that the person tries to continue the relationship in any way possible to avoid separation and being alone [58]. People who have other-directedness schemas have not been free to follow their own natural tendencies since childhood, and in adulthood, instead of being directedness from within, they are influenced by the external environment and follow the wishes of others in order to gain their satisfaction and approval. The evolutionary root of these people’s schemas has been based on conditional acceptance. Therefore, ambivalent people who have a stronger other-directedness schema, emphasize the answers of others more than their own needs in social relationships, they leave their control in the hands of others and submit to them. They obey others to avoid anger, revenge, or abandonment, and are always sacrificing to reduce the suffering and distress of others, avoid guilt, achieve a sense of worth, and continue the emotional relationship [49, 56]. This schema overlaps with the twelve-step concept of pathological dependence, and with the increase of the other-directedness schema, the experience of loneliness in people decreases [32].

### Implication for practice

Getting to know the basics of attachment and the quality of the first relationships in life in the formation of the EMS in people and reviewing this process from childhood to adulthood, along with explaining how it is related to the experience of loneliness, gives psychologists and therapists a more detailed and deeper look. Emphasizing how early relationships in life lead to the formation of EMS, this study provides some necessary elements to diagnose the causes of feeling alone and to create treatment protocols for working with young people, especially college students. This study provides psychologists and therapists with a suitable conceptual model for reframing the problems of people who complain of loneliness and seek treatment. Thus, based on the findings and explanations presented in this study, the concept that psychologists and therapists can focus on is the need for attachment and the mental framework formed from this need. One of the treatments that has been expanded and developed using the concepts of adult attachment theory as a method focused on the deep development of personality and its effect on the formation of intimate relationships is Emotional Focused Therapy (EFT). This approach was developed by Janson and Greenberg in the 1980s [67]. EFT treatment model considers emotions and feelings as key factors in shaping individual experiences and interpersonal interactions. In this approach, the therapist and clients look at the behavioral patterns and emotional experiences in people’s relationship with them and others and take action to create a more intimate bond and strengthen secure attachment in people and try to create a positive change in the individual’s emotional schema and the emotional pattern of relationships. Drawing attention to the emotional schema is the core of the EFT. In the sessions of therapy, the therapist tries to draw the emotional schema of the person in different situations and identify its repeating patterns and helps people to find new ways to react emotionally to the surrounding events [67]. The present study is one of the few that have shown this relationship between attachment, schemas and loneliness through the presentation of a structural equation model. The paths expressed in this study well show the role of attachment styles in the formation of schemas and the relationship between these schemas and loneliness. Moreover, presenting the concepts that were presented in the discussion section of the study provides new paths to the researchers of this field to take new steps in this field of study and cover the existing gaps.

### Limitations of the present study

One of the limitations of this study was the method of data collection, which was obtained through self-assessment questionnaires. Although these questionnaires provide useful information, sometimes they can reduce the validity of the obtained results. Also, although structural equation modeling has been used in this study, the nature of the relationships obtained is relational and not causal. In addition, this study was used on a sample of Iranians, and due to the existence of deep cultural differences in Eastern and Western societies, extreme caution should be exercised in generalizing these findings to other societies. Also, due to the peak of the Corona epidemic, the sampling method was convenience and online, which may distort the obtained data.

### Suggestions for future studies

Considering the limitations mentioned in the present study, it is suggested that future studies investigate the relationship expressed in this study using very accurate tools and in an environment with control of disturbing variables and using random sampling. Also, future studies can use longitudinal designs to present the causal relationship between these psychological constructs. Finally, re-doing these types of studies in different cultural contexts helps to form more accurate and universal findings in this field.

## Data Availability

The datasets generated and/or analyzed during the current study are not publicly available due to some legal limitations imposed by ethics committee of Razi University, Iran, but are available from the corresponding author on reasonable request.
